# Tensions in ethics and policy created by National Precision Medicine Programs

**DOI:** 10.1186/s40246-018-0151-9

**Published:** 2018-04-17

**Authors:** Jusaku Minari, Kyle B. Brothers, Michael Morrison

**Affiliations:** 1 Uehiro Research Division for iPS Cell Ethics, Center for iPS Cell Research and Application (CiRA), Kyoto University, Kyoto, Japan; 2 Kosair Charities Pediatric Clinical Research Unit, University of Louisville School of Medicine, Louisville, KY USA; 3 Centre for Health, Law and Emerging Technologies (HeLEX), Nuffield Department of Population Health, University of Oxford, Ewert House, Ewert Place, Banbury Road, Oxford, OX2 7DD UK

**Keywords:** Precision medicine, Genomics, Consent, Insurance, Privacy, Sustainability

## Abstract

Precision medicine promises to use genomics and other data-intensive approaches to improve diagnosis and develop new treatments for major diseases, but also raises a range of ethical and governance challenges. Implementation of precision medicine in “real world” healthcare systems blurs the boundary between research and care. This has implications for the meaning and validity of consent, and increased potential for discrimination, among other challenges. Increased sharing of personal information raises concerns about privacy, commercialization, and public trust. This paper considers national precision medicine schemes from the USA, the UK, and Japan, comparing how these challenges manifest in each national context and examining the range of approaches deployed to mitigate the potential undesirable social consequences. There is rarely a “one size” fits all solution to these complex problems, but the most viable approaches are those which take account of cultural preferences and attitudes, available resources, and the wider political landscape in which national healthcare systems are embedded.

## Introduction

Across the globe, governments are promoting precision medicine (PM) through national initiatives. High-profile examples include the 100,000 Genomes Project in the UK and the All of Us Research Program (formerly known as the Precision Medicine Initiative Cohort Program) in the USA [1, 2]. These state-supported endeavors aim to realize the potential of genomics and other data-intensive biomedical technologies to improve the accuracy of diagnosis, prevention, and treatment in clinical care. These national programs involve collecting data, building infrastructure, and constructing organizational arrangements to share this data, and to a lesser extent, building capacity among physicians, nurses, and genetic counselors to deliver PM services.

Several important challenges to the implementation of PM have been identified and widely discussed [3–6]. Notable concerns include those related to privacy, data protection, insurance, genetic discrimination, and the management of unanticipated results whose clinical significance is uncertain. However, with the possible exception of the well-publicized divergence on so-called incidental or secondary findings between the American College of Medical Genetics and Genomics and its European counterpart [7–9], many discussions consider these issues at a general or theoretical level rather than at a practical policy level. In particular, relatively little work has been undertaken to compare the approaches taken by different countries. This is an important gap in the literature, since national strategies for the translation of PM necessarily involve a range of trade-offs with respect to ethical and policy matters. Given that the implementation of PM potentially blurs traditional boundaries between research and clinical care in ways that challenge established models for consent, participation, social justice, and sustainability, it is important to consider these issues in real-life contexts.

The aim of this article is to compare the emerging strategies for translating PM, including learning healthcare system approaches, across three countries: the UK, the USA, and Japan. We focus on “precision medicine” as a topic that encompasses personalized, or at least highly stratified [10, 11], approaches to prevention, diagnosis, and treatment of diseases. Although many technologies might be used to inform such approaches, this analysis concentrates on genomic sequencing technologies, since this is a major component of all three national strategies we will examine. The USA and UK are each implementing significant national PM initiatives, but in the context of strikingly different healthcare systems. Japan’s PM program is in an earlier stage of implementation but represents an important non-Western example of such an initiative. All three countries have different legal and regulatory frameworks and different cultural backgrounds, although there is some evidence of growing convergence in public attitudes to linking and sharing of health data for research and communication between physicians and patients [12, 13]. It is not feasible within the scope of this paper to review the full scope of differences that could potentially affect the design and implementation of PM initiatives in each country. Instead, this analysis will begin by outlining the current approach taken by each country to promote PM. We then examine a number of major areas where PM presents key ethical, regulatory, and policy challenges and discuss how each national strategy has dealt with these challenges and what this reveals about their strengths and limitations. These challenges naturally involve, and therefore highlight, relevant aspects of each country such as healthcare systems, policy-making apparatus, and public attitudes and opinions that affect the implementation of PM in each territory. Ultimately, by examining these considerations across three distinct countries, we hope to identify key ethical, cultural, and regulatory factors that can inform similar initiatives in other countries and perhaps even support efforts to harmonize policies and ethical norms on an international scale.

## National strategies for precision medicine

### UK

The UK’s flagship PM initiative, the 100,000 Genomes Project, was announced in 2012. In 2013, Genomics England was founded to organize and coordinate the planned work of sequencing 100,000 genomes from around 70,000 patients in the UK’s National Health Service (NHS). Genomics England is configured as a company but is wholly owned by the UK Department of Health. The company is managed by a board that includes several prominent UK scientists. Patients are recruited through 13 designated NHS Genomic Medical Centers located across England. Participation from the UK’s other constituent regions—Scotland, Wales, and Northern Ireland—is subject to various local arrangements. More than 30,000 participant genomes are reported to have been sequenced. According to the Chief Medical Officer’s 2016 annual report, the desired outcome of the government’s PM programs is to transform the NHS into a model of a learning healthcare system with “research and care being alloyed together so that each activity is dependent on the other” (Chapter 16 page 9) [14]. However, the same report also observes that reaching this goal will require changes to several elements of the traditional social contract between healthcare professionals and the public including the meaning and function of consent, how uncertainty and contingency are dealt with in medical encounters, and how health data is collected, stored, and used (or not used). The UK government has currently committed to fund the venture through 2021.

Academic and other public sector researchers can access data from the 100,000 Genomes Project by joining one or more consortia known as Genomics England Clinical Interpretation Partnerships (GeCIPs). While the 100,000 Genomes Project focuses on two main disease areas—cancer and rare disease—each GeCIP is dedicated to a specific aspect of one of these disease areas, for example, “ovarian and endometrial cancer” or “pediatric rare disease.” Genomics England also coordinates with the UK Genetic Testing Network (UKGTN), which assesses single-gene tests for commissioning on the NHS, to share knowledge about existing variant-disease associations. In addition, Genomics England is set up to facilitate partnerships with private companies. Illumina (San Diego, USA) is the project’s official sequencing partner, while several other companies including Congenica (Cambridge, UK) and the multinational WuXi NextCode are also involved in developing tools for analyzing and interpreting the sequence data and associated health records. Genomics England’s strategy involves exploring the utility of different bioinformatics platforms through a series of “test phase” contracts where different firms work with a subset of sequence data from the project to demonstrate the capacities (and limitations) of their platforms [1].

Genomics England also operated a 2-year program (2015–2017) known as the Genetics Expert Network for Enterprises (GENE) consortium that brought together academics, NHS Genomic Medicine Centers, and private sector partners from the biotechnology and pharmaceutical sectors. Unlike the bioinformatics partnerships, GENE focused on the development of new diagnostics and treatments based on PM data from the 100,000 Genomes Project. The aim was to facilitate upstream engagement with commercial partners to ensure that the development and eventual outputs of the 100,000 Genomes Project are compatible with industry needs, which explains the relatively short-term nature of the collaboration. Further industry partnerships are reportedly planned.

### USA

The Precision Medicine Initiative (PMI), which was announced by then President Barack Obama in 2015, was originally envisioned as a multi-faceted research program focused on PM. The facet of this program that has received the greatest attention, however, is the All of Us Research Program, a biorepository and cohort study that plans to enroll over one million Americans. Coordinated and managed through the NIH Office of the Director, the All of Us Research Program pursues its recruitment, enrollment, data storage, and biosample collection and storage efforts through grants and contractual arrangements with corporations, non-profit organizations, healthcare systems, and universities. The program is currently developing two methods for recruiting and enrolling participants. In the first, eight healthcare provider organizations will recruit participants from their patient populations. In the second, individuals will volunteer to participate online and then visit a retail pharmacy or other contracted location to have their blood or other biosamples collected. The approach to making data and biosamples available to researchers is currently in development, but the PMI has expressed its intention to make these resources widely available to both academic and commercial investigators. Funding for the PMI, including the All of Us Research Program, is determined by the US Congress as a part of its overall budget process. Given the political nature of this process, the potential for long-term funding of the All of Us Research Program remains unknown, although it has received continued support in the transition from the Obama administration to the Trump administration [15].

The All of Us Research Program is just one of a number of federally funded efforts in the USA to develop PM. Another dimension of the PMI focuses on developing precision medicine to treat cancer, as does a related effort termed the “Cancer Moonshot.” The Electronic Medical Records and Genomics (eMERGE) Network started out as a federally funded network of biorepositories but has more recently evolved into a program that also focuses on delivering predictive genomic research results into clinical care. The Clinical Sequencing Evidence-Generating Research (CSER2) Consortium, funded by the National Human Genome Research Institute (NHGRI) and the National Cancer Institute (NCI), is designed to integrate genomic sequencing tests into the routine practice of medicine, including in the diagnosis and treatment of rare diseases [16].

In their current iterations, none of these national efforts involves full-fledged implementations of a learning healthcare system. The All of Us Research Program, the eMERGE Network, and the CSER Consortium all involve strategies for returning findings from genomic sequencing to research participants and their healthcare providers, with research aims designed to observe how these results affect clinical care and clinical outcomes. However, these efforts are not yet designed to create the feedback loop between clinical care and research envisioned for the learning healthcare system model.

### Japan

In Japan, the government has responded to the advent of an aging population by launching a healthcare innovation initiative to ensure a healthy, long-living society [17]. In 2014, two acts regarding healthcare innovation policy were passed: the Act on Promotion of Healthcare Policy and the Act on the Independent Administrative Agency of Japan Agency for Medical Research and Development. These acts led to the establishment of the Headquarters for Healthcare Policy (HHP), which is located in the Cabinet^1^, and the Japan Agency for Medical Research and Development (AMED), as a funding agency attached to three ministries. The Headquarters for Healthcare Policy provides a central organizational hub to strategically promote healthcare innovation, while AMED’s goal is to “accelerate the seamless and cooperative translation of basic research to clinical application” through the award of research grants. The mandate for these organizations signals that Japan’s nationally driven healthcare initiative incorporates the realization of genomic medicine as one of its key goals. As a part of this initiative, the Council for Realization of Genomic Medicine (CRGM), which is composed of representatives from cabinet secretariat, ministries, agencies, academia, and others, was established in 2015. The purpose of the council is to consider and present a specific national vision and approach to realize genomic medicine. Although an operational learning healthcare system has not been addressed yet, several initiatives to collect genomic data and connect this with electrical health records have been promoted, especially in relation to cancer.

AMED which “has a wide-ranging mandate to smooth the flow of basic discoveries to the clinic and the market” is promoting three research projects [18]: “Platform Program for Promotion of Genome Medicine” (a 5–10-year project for common diseases, in coordination with the Ministry of Education, Culture, Sports, Science and Technology (MEXT)), “Program for an Integrated Database of Clinical and Genomic Information” (a 3–5-year project for rare disease, cancer, and others with the Ministry of Health, Labor and Welfare (MHLW)), and “Program for Promoting Platform of Genomics based Drug Discovery” (a 3-year project for clinical implementation with MHLW). AMED also established a genomic data sharing policy in 2016. As a result, the sharing of genomic data is increasingly required for Japanese databases. For example, the NBDC Human Database is designed with both a managed/controlled system and an open access system. Several large-scale biobanks have been provided with ongoing support, including the Tohoku Medical Megabank Organization (ToMMo). This program has collected samples from more than 150,000 healthy people and analyzed a few thousand whole genome, high-coverage sequences. This resource has been used to estimate the frequencies of actionable pathogenic variants (as specified by the ACMG) in the Japanese population [19], and the active utilization of this and other biobanks for broader stakeholders is being strongly encouraged.

Another relevant activity, the Initiative on Rare and Undiagnosed Disease (IRUD), was launched in 2015 to “maximize the benefit of whole-exome and whole-genome analyses for patients searching a diagnosis [20].” It has already achieved a registry of more than 2000 undiagnosed patients. In these research programs, collaborative efforts to utilize the existing sequencers located in universities, research institutes, and emerging sequencing companies are being strongly encouraged. Several elements of these efforts are starting to reach clinical care. For example, in 2016, significant effort was undertaken by the national healthcare services, expanding the number of rare diseases for which genetic testing is covered from 36 conditions to 72 conditions. In addition, the MHLW released a 2017 report representing a new direction in cancer genomic medicine. This will involve the identification of core centers of cancer genomic medicine in Japan, followed by the translation of genome panel testing into national healthcare services.

## Ethics and policy trade-offs

The areas of cancer and rare diseases, which are addressed by national programs in all three countries, are widely regarded as “low-hanging fruit” for this approach. Although the evidence base in these two domains is still developing, early findings indicate that the balance of risks and benefits created by PM may be favorable. In rare diseases in particular, simply improving the chances of providing a diagnosis creates a substantial benefit. However, many scientists, policymakers, and industry leaders in all three countries aspire to the development of PM as an entirely new model for the way medical science addresses a wide array of diseases and conditions, including areas where the risks and benefits of PM remain undefined. Given this aspiration, it is particularly important to consider carefully the opportunities and challenges that PM poses in the domains of ethics and policy. Potential trade-offs between opportunities and challenges in at least three overarching domains will prove particularly important to further efforts to implement PM: (1) genomic sequencing in the context of the learning healthcare system, (2) implications for healthy individuals, and (3) sustainability and private-public coordination. In the sections that follow, we examine the trade-offs in each of these domains.

### Genome-wide sequencing and the learning healthcare system

The concept of the learning healthcare system [21–24] envisions that both scientific insights from emerging technologies and the technologies themselves can be applied to clinical care on an ongoing, though flexible and contingent, basis. Although this framework is, in theory, applicable to any technology, PM efforts tend to emphasize the application of genome-wide sequencing (GWS)^2^ within learning healthcare systems. Research using GWS has started to generate knowledge in focused areas that can be useful for clinical practice [8], but this technology can also generate a wide array of results whose implications are not yet well understood. The learning healthcare system approach suggests that if GWS is utilized in clinical contexts, despite this incomplete knowledge, both scientific knowledge and clinical care will be improved. For genetic variants that are thought to be clinically actionable, the clinical application of GWS will supply evidence to assess this value. For genetic variants that are not well understood, national PM efforts provide an opportunity to collect genomic data and clinical phenotypes from populations that are more representative than those involved in previous case-control studies, thus improving the understanding of the penetrance and pathogenicity of these poorly understood genetic variants. As more reference genomes from healthy volunteers and patients with milder phenotypes enter databases, the clinical significance of these variants may be revised, leading to additional changes in clinical practice.

Taken as a whole, the learning healthcare system is based on the strategy of integrating information into clinical practice prior to the availability of clinical evidence for its benefit and perhaps even when it is uncertain whether this information is clinically valid or relevant. An inherent dimension of the learning healthcare system, then, is the idea that the evidence base for the utility of genomic sequencing and other technologies can be built by utilizing these technologies in practice and observing what happens. A central debate, both from a medical perspective and from an ethics and policy perspective, is whether this should be thought of as a “feature” of the learning healthcare system or a “bug.” On the one hand, much of the scientific value of this strategy lies in the opportunity to observe what happens when results that are currently uncertain are integrated into clinical care. On the other hand, this strategy inevitably involves the application of technologies to clinical care while their risks and benefits remain poorly defined. Learning healthcare system approaches, then, must also account for the potential risks created by feeding uncertain information into the clinical enterprise and must utilize strategies to prevent and reduce potential harms to patients. Moreover, in all three case study countries, the separation between research and clinical care is inscribed in current legal instruments and regulatory systems.

The issue of risk created by returning uncertain information naturally leads to ethical and policy issues, primarily related to informed consent. In a translational research setting, the pitfalls associated with informed consent for GWS are already well-documented [25]. These include difficulty with anticipating and explaining all of the potential findings along with their associated risks and benefits. In the context of the learning healthcare system, these challenges multiply. Research and clinical care are blurred, potentially creating confusion for patients about whether GWS is being recommended to answer a research question or because the provider believes available evidence supports its use in the patient’s particular circumstances. In addition, because the learning healthcare system by design involves frequent changes to clinical management, the difficulty with providing a meaningful accounting of the risks and benefits of participation is increased immensely. Therefore, bridging the gap on informed consent between research and clinical care is potentially critical to the application of GWS in the learning healthcare system.

National PM programs, if they are to implement the learning healthcare system framework, will need to carefully consider these concerns. In the short term, many of these programs, such as the eMERGE Network in the USA and the 100,000 Genomes Project in the UK, have simplified this challenge by demarcating return of research results from clinical care. During a recent American Society of Human Genetics workshop, Genomics England chief scientist Mark Caulfield explained that although findings from the project are fed back to patients by their NHS clinician, the results are not regarded as a diagnosis that the clinician is obligated to return as part of the patient’s care [26]. 100,000 Genomes Project participants are also offered the choice to opt out of receiving information about “secondary” or additional findings (those not related to the condition that made the participant eligible to take part in the study). In the eMERGE Network in the USA, individual sites develop their own methods for returning results, although in general these approaches make it clear to participants that results are being provided as a result of the research study to which they had consented and not as a part of their routine clinical care. In the longer term, national efforts to implement a learning healthcare system will need to pursue process innovations that adapt informed consent and return of results to this dynamic context [12, 27–29], and further develop appropriate systems of regulations, governance, and oversight adapted to the learning healthcare system framework.

### Considerations for healthy individuals

Recruitments of healthy people have several advantages for research. At present, the USA is aiming to incorporate large-scale analysis of genomic and health data from healthy people as part of its national PM initiatives, and Japan is also promoting WGS for a considerable number of healthy people. As noted above, analysis of healthy people provides a reference point for comparing data from patients with various conditions as a means of evaluating the penetrance and pathogenic effect of variants. Sequencing asymptomatic individuals also creates opportunities for the early detection of disease risk, pharmacogenetics-informed prescribing, reproductive decision-making, and counseling on preventive health strategies. Long-term population cohort studies can provide valuable insight into the genetic contribution to the development and progression of diseases. In addition, research on the perspectives of research participants indicates that participation in medical research can be beneficial in a number of ways, including satisfaction from contributing to future public benefits.

Many contemporary PM initiatives, including those pursuing the learning healthcare system model, offer healthy participants the opportunity to obtain individual genomic data, including “incidental findings” with potential clinical significance. Recent research suggests that a small, but significant percent of healthy participants might harbor clinically actionable variants associated with significant conditions [19, 30, 31]. This emerging practice is a significant departure from past research, where it was uncommon to return individual genetic results to healthy research participants.

These changes in research on healthy volunteers will create new tensions between benefits and risks in PM research [31–33]. Although the genetic results returned to healthy participants in the national programs we have highlighted are typically limited to “actionable” genomic variants, the outcomes from reporting these findings remain unknown. Actions taken because of an unexpected result are likely to expose participants to risks they would not have encountered in routine care (such as additional radiation exposure due to imaging). This is critical, since a significant proportion of the individuals who have these variants will in fact never develop the associated conditions. This is especially problematic for variants that are poorly understood, where the likelihood that individuals will develop the associated conditions—the penetrance of the variants—is often overestimated [34]. Reporting genomic results to patients can also create anxiety, fear, or confusion, with patients left wondering when and if they may develop the associated condition. Therefore, returning those results to healthy volunteers should be carefully considered, and when returning following ethical and legal requirements, those results should not be regarded as conclusive data but as supportive or reference data for clinical decision-making at least in the near-to-medium term.

There are also important questions about who else should receive this information including insurance companies, employers, and relatives. The detection of variants of unknown or uncertain significance could carry ramifications for life insurance or other services. For healthy individuals, altered eligibility for insurance based on this information may be perceived as unwelcome and unjust. At the same time, people who discover previously undetected health risks because of PM initiatives may be incentivized to purchase additional insurance if uncertainty over the status of genomic findings means they do not have to disclose that information to insurers [35]. Several nations have already adopted specific legal provisions to address concerns about insurance [36–40]. In the USA, the *Genetic Non-Discrimination Act*, often referred to as GINA, prohibits insurers from utilizing genetic information in health insurance decisions, including the setting of insurance rates, but there is no prohibition against this practice in life insurance or long-term care insurance. For most European countries, basic healthcare coverage is provided by the state, but life insurance and other coverage is not and must still be purchased from private providers. The UK (along with Germany and the Netherlands) has a voluntary moratorium on the use of genetic data in calculating insurance premiums. Most other European countries have opted for formal legal prohibitions. In contrast, most East Asian countries have not implemented these kinds of specific legal regulations [36–38, 41]. In Japan, there is ongoing debate about whether a specific law on genetic discrimination is needed, especially given the existence of other legal instruments regulating the protection of personal information and prohibiting unjust discrimination by the insurance industry.

It is also important to consider effects on family members [29, 42–44]. When a variant with potential clinical implications is detected in one person, it potentially has implications for the provision of care to relatives. This issue also arises with some existing practices, such as when a clinical diagnosis of breast cancer is made, but the prospect of implementing PM in routine care will constitute a massive expansion of tests that require healthcare professionals to consider family health as well as care of individual patients. If sequenced individuals do not wish to share these results with family members, this can create “a conflict of normative duties and values: respecting individual confidentiality and autonomy on one and preventing potential harm to a relative on the other [44].” While the appropriate measures for addressing this challenge may vary from country to country, in the countries selected for our case studies, there are no legal instruments that unequivocally establish a duty for healthcare providers and researchers to disclose genomic information from family members. In the UK, non-binding guidelines advocate that patients voluntarily disclose genomic and other medical data when it has implications for the health of relatives but permit physicians to disclose confidential medical data to a patient’s relatives even in the absence of consent if the benefits of disclosure clearly outweigh the risks [45]. Non-binding guidelines in the USA similarly support the sharing of genetic information with family members when this could be helpful. However, the provisions of the Health Information Portability and Accountability Act (HIPAA) arguably prevent the disclosure of a genetic finding to family members if the patient objects [46]. In Japan, non-binding governmental guidelines stipulate that priority be given to ensure consent by research participants, but there are also exemptions to enable healthcare providers to disclose genomic results to family members where consent has not been given for their disclosure [47]. Again, these exemptions exist where disclosing the information is likely to prevent serious damage to the wellbeing of the people affected by the disclosure.

As national efforts to explore the learning healthcare system model expand, the challenges related to disclosing genomic findings to healthy people and their family members will become more common. Providers will more frequently face questions about how to balance the (sometimes conflicting) obligations of participant confidentiality and a “duty to rescue” family members from genetic risk. They will also face questions about privacy and discrimination. Although policies and regulations to provide protections related to privacy and discrimination have been implemented in all three countries, there is substantial evidence that these policies and regulations do not necessarily assuage the concerns of patients and research participants [39, 48–50]. Ultimately, decisions about how to address these challenges will need to be based on a number of contextual factors, including the structure of research and healthcare systems, available resources, cultural preferences and attitudes, and government priorities.

### Sustainability and private-public coordination

Implementing PM in routine practice is likely to involve significant costs beyond those associated with single-gene tests. In the USA, access to single-gene testing is determined by insurance coverage, while in the UK and Japan, provision of specific tests is governed by national healthcare systems. In particular, the UK case, where each potential new test is assessed by the UK Genetic Testing Network in terms of potential to reduce mortality/morbidity and the impact of offering testing on existing services, illustrates that cost and resource management is already a factor in this domain. While the cost of a high-quality GWS for an individual has dramatically fallen due to the development of next-generation sequencers, national PM initiatives are likely to incur a range of other costs, not least developing and maintaining the necessary digital and physical infrastructure to manage samples and data [51]. Government funding is often organized around specific projects or missions and is generally for a fixed period. Long-term funding depends on a range of factors, including evaluations of earlier work performed, the perceived importance of the activity being supported, and other strategic and economic concerns. The sustainability of PM efforts, including biobanks, registries, and other types of infrastructure, is a recurring challenge [52–57].

Greater private sector involvement with PM is one way to address the challenge of sustainability for national initiatives. Public-private partnerships offer one mechanism for facilitating commercial access to samples and data on a non-exclusive basis. A commercial firm can access the samples and data held in a public biobank or repository for purposes such as validating existing biomarkers or developing diagnostic, prognostic, or pharmacogenetic tests without inhibiting future access through ownership or intellectual property claims [55]. However, public-private partnerships for PM, which involve the use of samples and data that were obtained through public institutions using public funds, can also create a number of major ethical and social challenges, especially related to participants’ trust in research and concerns about privacy.

Although is often assumed that the involvement of biomedical or pharmaceutical companies in research will raise concerns about privacy, there is evidence that it is not simply the presence or absence of industry partners that affects public opinion. Rather, the type of private firm and the nature of their involvement are important. Several recent surveys found that a slight majority of respondents would be in favor of sharing health data with commercial companies if they could envisage a benefit, such as better medicines, while access to data for insurance and marketing was not well-received [13, 58]. Balancing the involvement of private interests is critical. Involving companies in ways that decrease public trust may also lead to reduced rates of participation and reduced willingness for broad data sharing [59, 60].

A range of different models for private involvement has been explored. Genomics England employs a direct contractual approach for its bioinformatics partnerships. In its contracts for sequencing (Illumina) and analytic (WuXi NextCode and others) services, payment is provided on a pre-negotiated fee-for-service basis, and other benefits to the companies are contractually limited. Illumina, for example, does not own any of the sequence data generated, and the company does not get any access to non-genomic patient data. The GENE consortium, in contrast, utilizes a public-private partnership model. Commercial partners are required to pay a fee to join the consortium and in return are granted access to a subset of aggregate sequence data and de-identified patient records. The research, carried out collaboratively by industry, academic, and NHS members, is positioned as a pre-competitive space. Participating partners have certain contractually mandated rights and obligations; for example, all results of the research must be published, but this can be delayed to allow intellectual property claims arising from the research to be secured.

These lessons from the UK demonstrate only a small sample of the numerous funding strategies that can be utilized to move PM forward while laying the groundwork for future sustainability in the private sector. In the USA, for example, Geisinger Health, a regional healthcare system, has established a contractual arrangement with Regeneron, a pharmaceutical company. Through this arrangement, GWS data is combined with electronic healthcare data to create a significant PM resource. As a result, Geisinger is able to pursue academic research, often with federal research funding, while Regeneron is able to pursue the identification and development of new drug targets [61]. While governmental funding approaches have previously been a cornerstone of genome research, public-private partnerships can contribute to achieving sustainable PM. This means, however, that while the privacy of biosamples and data providers will need to be carefully managed, the sharing of benefits among stakeholders will also need to be adequately facilitated through prospective contractual arrangements.

## Conclusions

The above discussion has highlighted a number of challenges facing the successful implementation of PM at the national level. This is by no means an exhaustive list. We have focused on ethical and social challenges that arise from the “real world” implementation of national PM programs. Conducting research, even with the most laudable of aims, requires a “social license” in terms of ensuring that both the conduct of the research and its impact are in line with widespread ideas of what is desirable, fair, and appropriate. The scope and scale of implementing PM, especially as part of a learning healthcare system, is such that securing a social license requires attention to multiple dimensions: public trust, financial viability and sustainability, legal and regulatory considerations, organizational factors, and issues of equity and social justice. PM ultimately involves developing new ways of classifying people and groups. In order to reap the benefits of these new classifications, it is therefore important to ensure that appropriate structures are in place to mitigate or prevent any potential negative outcomes of these new taxonomies.

The comparative effort we have undertaken in this paper provides some useful insights, but it is only a first step. A range of significant issues remains in need of analysis including ownership and other legal rights related to genomic data [62], the distribution of benefits among patient communities who contributed data to PM research, and the potential relationship between more “precise,” but less homogenous, treatment strategies and pay-for-performance payment models. Furthermore, this variation between nations will likely create challenges for the global harmonization of PM efforts that will need to be explored further in future work. For example, the sharing of biosamples across international boundaries and the use of cloud computing systems to share and analyze genomic data on an international scale both raise ethical and regulatory concerns about privacy and security, which can be also deeply associated with the policy and direction of national data infrastructures [63]. In order to address these issues, the role of local data access committees and the limitations of global regulations and oversight systems will need to be explored further [64, 65]. Our comparison of three countries makes it clear that the global PM community still has a great deal of work to do.

## Footnotes

1 In Japan the Cabinet is the executive branch of the government and consists of the Prime Minister and senior officials known as Ministers of State.

2 Genome-wide sequencing (GWS) includes whole genome sequencing, whole exome sequencing, and other technologies that sequence large regions or panels of genes. The UK primarily focuses on gene panel technologies, while the USA and Japan put greater emphasis on whole exome or whole genome sequencing for both healthy individuals and patients.
